# The impact of Covid-19 on research into work and health

**DOI:** 10.1093/occmed/kqac080

**Published:** 2022-08-30

**Authors:** V Parsons, E Wainwright, M Karanika-Murray, G Muiry, E Demou

**Affiliations:** Occupational Health Service, Guy’s and St Thomas NHS Foundation Trust, London SE1 7NJ, UK; School of Life Sciences and Medicine, King’s College London, London SE1 7UL, UK; UK Medical Research Council Versus Arthritis Centre for Musculoskeletal Health and Work, University of Southampton, Southampton SO16 6YD, UK; UK Medical Research Council Versus Arthritis Centre for Musculoskeletal Health and Work, University of Southampton, Southampton SO16 6YD, UK; Epidemiology Group, School of Medicine, Medical Sciences and Nutrition, University of Aberdeen, Aberdeen AB25 2ZD, UK; Psychology Department, Bath Spa University, Bath BA2 9BN, UK; Department of Psychology, Nottingham Trent University, Nottingham NG1 4FQ, UK; Occupational Health Service, Guy’s and St Thomas NHS Foundation Trust, London SE1 7NJ, UK; MRC/CSO Social and Public Health Sciences Unit, University of Glasgow, Glasgow G3 7HR, UK

## Abstract

**Background:**

The global coronavirus (Covid-19) pandemic created a profound disruption to the delivery of planned scientific research with unknown immediate and potentially longer-term impacts.

**Aims:**

We explored researchers’ experiences of the impact of the pandemic on the continued development and delivery of research into work and health, and on research infrastructure in this field.

**Methods:**

A cross-sectional study.

**Results:**

Thirty-three questionnaires were completed, representing a response rate of 15%. Sixty-one per cent of respondents were female, the majority (78%) had over 11 years of research experience and 76% worked mainly in academia. Most respondents (88%) were able to progress with research during the pandemic. A small proportion (4%) had studies paused or suspended due to the pandemic, while a larger proportion (19%) had research staff redeployed to assist with other studies or furloughed. Respondents described a range of emerging practical and logistical issues for research into work and health during the pandemic. Some benefited from increased opportunities to collaborate on new multidisciplinary studies, opportunities to engage participants in work and health research, and more flexible and inclusive work practices. Others experienced challenges that had an adverse impact, such as hampering research delivery (e.g. barriers to participant screening and intervention delivery), poor (home) working environments, reduced team cohesion and isolation. A range of future priorities for research was highlighted.

**Conclusions:**

We describe lessons learned and opportunities that can be used to support or further research activities in the field of work and health research in the future.

## Teaser

The Covid-19 pandemic had a significant impact on research activity across many disciplines and diverse areas of health and medical research. It also provided opportunities for researchers to explore new and novel areas of research beyond traditional areas of interest. This study explored the impact specifically on work and health research planning and delivery, and proposes future research priorities which are likely to follow as we emerge into an endemic world.

**Key learning points**

What is already known about this subject:

- Research into work and health is a broad and diverse field, with researchers representing different disciplines and specialties (medicine, public health, psychology), and employment sectors such as academia, industry.
- For some researchers, the Covid-19 pandemic had an abrupt impact on the planning and delivery of established health research although its impact on research into work and health was unknown.
- In some sectors such as healthcare, regulators, funders and research sites imposed strict mandated requirements which prioritized Covid-19-related research activity. This included the temporary suspension of non-Covid-19 studies and the diversion of research resources. In other sectors, the progression of research into work and health was less affected.

What this study adds:

- This was the first study to explore the impact of the current pandemic on the continued development and progression of research into work and health. We identified a number of novel gains and opportunities which were created for researchers during this unprecedented period.
- We found that agile ways of working (e.g. such as remote working) and harnessing online technology created a more inclusive research environment for researchers and participants, offered greater flexibility and autonomy, and opened up new collaborative opportunities.
- We highlighted emerging research priorities which are likely to shape the future of research into work and health. Examples included the impact of Covid-19 on respiratory health and work functioning, digital health interventions for chronic diseases and the impact of remote working on mental and physical health and disabilities.

What impact this may have on practice or policy:

- In further developing academic research, it is possible to highlight priorities for research into work and health, and areas where a level of knowledge integration will be useful. It is important to identify specific research topic areas and populations that may become vulnerable due to a lack of needed research.
- In terms of developing support and building research capacity, networks can be created of researchers in these priority areas to preserve capacity and more readily deploy expertise. At the same time, mechanisms to rapidly mobilize researchers should be developed and scaled up.
- Policy implications include safeguarding the sector from ‘shocks’ to resources and capacity, redirecting resources to support precarious fields of research, future research priorities, and protecting opportunities for early career researchers. Efforts to support research into work and health would protect the UK’s research rigour and reputation in this field.

## INTRODUCTION

In response to the evolving Covid-19 pandemic, the Department of Health and Social Care, national regulatory bodies and local health and academic institutions in the UK published rapid guidance for researchers [1,2]. This guidance was intended to safeguard the scientific integrity of health research during the pandemic, and critically to direct finite resources towards supporting urgent Covid-19-related studies. They were consistent with international guidance [3–6]. Furthermore, research with employees/employers in all workplaces was also impacted, although research in other sectors was less adversely affected [1,7]. This disruption gave rise to complex ethical challenges regarding participant participation, and safety and welfare issues which required risk mitigation [7,8]. In late 2020, this period was followed by a coordinated restart and prioritization programme of clinical non-Covid-19 studies across the UK.

Various studies have explored the impact of the Covid-19 pandemic on research across different fields of health research [5,9–11]. A study focusing on six research projects at one healthcare institution in the USA found that there had been a considerable decrease in the number of eligible participants approached and consented into non-Covid-19 studies, despite the availability of research staff to support their continuation. The authors attributed this decrease in recruitment to their inability to approach eligible participants [9]. Researchers also identified gender disparities in research activity during the pandemic, in particular, a decrease in publication output among females with caring responsibilities [12,13].

Despite these challenges, the pandemic stimulated new opportunities for researchers, including opportunities to collaborate beyond traditional research domains as research interests shifted to investigating novel aspects of the SARS-CoV-2 virus and its impact on workers and the workplace [8,14].

While the literature highlights some of the practical challenges of delivering research during the pandemic [15], work describing the consequences of the Covid-19 pandemic for research in the field of work and health (e.g. interventional studies and occupational epidemiological research) is currently unavailable. Therefore, the aim of this study was to assess the impact for the continued development and delivery of research into work and health during the pandemic period, and to identify potential priorities in this area of research which are likely to follow in the future. This study targeted researchers representing a broad range of disciplines.

Our study focused on the following questions:

1. What impact has the Covid-19 pandemic had on the continuation of research into work and health?
2. What opportunities have arisen to optimize the development and delivery of research into work and health in the UK during the Covid-19 pandemic?
3. What lessons could be learned to safeguard and facilitate the progression of rigorous and high-quality research into work and health should similar disruptions to research occur in the future?
4. What priorities for research into work and health are likely to emerge in response to the Covid-19 pandemic?

## METHODS

This study comprised an anonymous online survey targeting work and health researchers in the UK. The survey was open for completion between 28 October 2021 and 9 January 2022. Several e-mail reminders and alert notifications were used to optimize the responses, as per recommendations [16].

We used purposive and snowballing sampling methods. Specifically, we identified potential respondents by reviewing lists of work and health-themed projects publicly available from UK-based funding bodies using common search terms, e.g. ‘workplace interventions’, ‘workplace health promotion’, ‘occupational’. We then sent study information to study leads where contact details were available and to funding bodies with a request that they promulgate this information to grant holders. We also disseminated study information among our professional networks.

No personal identifiable information was collected. All participants read the Participant Information Sheet and provided consent, in line with ethical guidance on internet-mediated research [17]. Approval granted by Bath Spa University Ethics Committee. The survey was piloted twice with stakeholders who did not suggest any major changes.

The questionnaire covered: (i) demographics and information on involvement in work and health research projects during the Covid-19 pandemic period; (ii) feedback on practical challenges and enablers with regard to research activity and delivery and (iii) views on future research priorities which are likely to follow in response to the Covid-19 pandemic.

We used descriptive analysis and presented the results as proportions and frequencies of the total number of all responses. Qualitative free-text data were analysed by themes using our predefined research questions as a guide.

## RESULTS

Study information was sent to approximately 226 organizational and individual contacts. From this, we received 33 completed questionnaires. We expect that a much larger number of individuals received the questionnaire, as per the snowballing method. On the basis of the initial reach, the number of responses represents a 15% response rate, which is acceptable for online surveys [18]. An overview of the respondents demographics is outlined in Table 1.

**Table 1.** Respondents’ demographic characteristics

	*n* (%)
Gender	
Male	10 (30)
Female	20 (61)
Prefer not to say	1 (3)
Non-binary	1 (3)
Missing	1 (3)
Years of experience	
0–10	7 (21)
11–20	14 (42)
21–30	7 (21)
>31	5 (15)
Stage in career	
Established researcher	21 (64)
Mid-career researcher	7 (21)
Early career researcher (within 5 years post-PhD)	3 (9)
Not applicable	2 (6)
Job role	
Academic	25 (76)
Clinical academic	4 (12)
Management and administration	2 (6)
Clinically trained emeritus academic	1 (3)
Clinician	1 (3)
Research role	
Principal investigator	18 (55)
Co-investigator	16 (48)
Research manager	4 (12)
Study manager	1 (3)
Postdoctoral researcher	6 (18)
Honorary research fellow	1 (3)
Research associate	1 (3)
Employment sector (select all that apply)	
University	29 (85)
Private sector	2 (6)
National Health Service (NHS)	5 (21)
Public sector (non-NHS)	2 (6)
Funding body	2 (6)
Voluntary	1 (3)
Retired and Emeritus at University	1 (3)
Freelance	1 (3)
Core discipline	
Psychology	12 (36)
Occupational health	5(15)
Occupational therapy	
Social work	1 (3)
Medicine	5 (15)
Nursing	2 (6)
Rehabilitation	1 (3)
Statistics	2 (6)
Physiotherapy	1 (3)
Health economics	1 (3)
Epidemiology	1 (3)
Social history, social care policy	1 (3)
No answer	1 (3)

Most respondents were employed in the university sector, of which 15% had dual roles in the National Health Service. The respondents reported specialist knowledge in a range of diseases and health conditions, areas of research interest and methodologies, e.g. clinical trials, Covid-19 secondary analysis, disabilities, epidemiology, ergonomics/job redesign, exposure assessment, knowledge transfer, long-term health conditions, musculoskeletal conditions, occupational psychology and psychiatry, occupational respiratory diseases, pain, primary care, psychometric testing of work outcomes, return to work, rheumatology, sickness absence, workplace well-being and work attrition.

The majority of respondents (88%; *n* = 29) reported continued development and progression of research into work and health during the pandemic, e.g. grant development and strategic research work, with a larger proportion (97%; *n* = 32) also undertaking research delivery activities, i.e. site set-up, participant recruitment, intervention delivery, follow-up and data analysis. Additionally, the number of new or ongoing non-Covid-19 work and health research projects which respondents were involved in since the start of the pandemic ranged from 0 to 10 projects (*M* = 4, SD = 2.4); while their involvement in Covid-19-related research projects relating to work and health during the same period was lower, i.e. 0–6 projects (*M* = 2, SD = 1.8). On average, respondents reported that the Covid-19 pandemic had a moderate impact (*M* = 7, range: 1–10, SD = 2.0) on the continuation of their research activities.

We found respondents were involved in a diverse range of research projects during this period, with the majority comprising primary research (interventional studies) and epidemiological and secondary data analysis research (Table 2).

**Table 2.** Overview of main fields of research into work and health research activity during the pandemic

• Covid-19	• Development of consensus guidance
• Digital health interventions	• Disability
• Data linkage, epidemiological and secondary data analysis research	• Health interventions
• Health and well-being at work	• Healthcare policy
• Health economics	• Health surveillance
• Mental health and occupational psychiatry	• Mindfulness
• Multiple long-term conditions	• Musculoskeletal disorders
• Older workers	• Pain
• Physical measurements	• Psychometric testing in the workplace
• Psychosocial issues	• Rare bone diseases
• Respiratory health	• Return to work
• Sickness absence	• Systematic reviews
• Work attrition	• Work rehabilitation
• Workplace exposure assessment	• Workplace safety

As highlighted in Table 3, a small proportion of projects were either paused/suspended due to the pandemic, with a higher proportion able to progress to study delivery and completion throughout this period. Seventeen per cent of respondents were unable to complete their projects as planned. Similarly, we found only 19% of respondents experienced staffing implications (redeployment and furloughing research staff) during this period.

**Table 3.** Impact and implications of Covid-19 on progression of research into work and health

	*n* (%)
During the Covid-19 pandemic period, were you required (by funder or employer to pause or suspend your project, e.g. due to recruitment issues or infection control concerns?	
Yes	8 (4)
No	43 (23)
Unsure	1 (0.5)
What stage did you reach in the research project timeline?	
Grant development	2 (4)
Study set-up	3 (6)
Study delivery	11 (21)
Study completion	5 (9)
Did the funder require (or support) a variation to the contractual arrangement terms?	
Yes	6 (11)
No	11 (21)
Not applicable	2 (4)
Unsure	2 (4)
If you selected ‘Yes’, please specify	
No cost extension	5 (9)
Reduction in original funding amount	1 (2)
If applicable, did the project partner (e.g. workplace) require (or support) a variation to the contractual arrangement terms?	
Yes	7 (13)
No	8 (15)
Unsure	3 (6)
What is the current status of this research project?	
Ongoing (never stopped)	29 (55)
Temporarily paused by research team	8 (15)
Recommenced	5 (9)
Completed	10 (19)
Did you complete this research project as planned (i.e. at write-up stage or work published)?	
Yes (write-up in progress)	17 (32)
Yes (work published)	8 (15)
No	9 (17)
Not applicable	15 (28)
During the Covid-19 pandemic period, were any staff working on this project redeployed to assist with other (research or non-research) duties or furloughed?	
Yes (redeployed)	8 (15)
Yes (furloughed)	2 (4)
No	43 (81)
If redeployed, please specify:	
To support Covid-19 studies	4 (8)
To support other non-Covid-19 studies	2 (4)
To perform clinical work	4 (8)
To perform non-research duties	2 (4)

Respondents considered that several opportunities had arisen across different domains during the pandemic which had positive benefits for research activity. Most noteworthy were increased opportunities for researchers to establish new partnerships beyond their traditional research boundaries and collaborate on novel fields of research. Some relished this wider and more active engagement with new collaborators. Participants also reported that government and funding bodies gave greater recognition to the importance of research into work and health and for the need for better understanding the complex interaction between work and health. This created additional funding opportunities which may not have existed otherwise. Their involvement in the planning and development of Covid-19-related studies also created valuable opportunities for researchers to explore the impact of the Covid-19 virus on work-related issues and outcomes, and a chance for some to adapt ongoing non-Covid-19 studies. However, adapting existing non-Covid-19 studies at pace to ensure their sustainability also created challenges. One respondent highlighted the future potential benefits which are likely to follow from the surge in Covid-19-related research activity, particularly regarding the exponential increase in data relating to work and health which was collected during this period.

While the surge in Covid-19 research activity created exciting opportunities to broaden research interests, several respondents expressed concern about the quality and rigour of rapidly executed research studies, the duplication of Covid-19 research studies, the diversion of scarce research resources and vital funding to support Covid-19 studies at the expense of other fields of research, coupled with the deprioritization of non-Covid-19 research into work and health:

too much focus on the same covid-related (often exploratory) topics at the start of the pandemic (ie the impact of the pandemic on health/wellbeing etc) and startling duplication of work and waste of resources - due to the above, uncoordinated research

Some respondents considered the progression and delivery of research was aided by the adaption of efficient ways of remote working within research role, particularly the rapid implementation and use of digital technology (online platforms) which allowed for increased research planning and collaboration opportunities to take place by bringing geographical dispersed researchers together more readily and within a short period of time.

For some, this dynamic way of planning and delivering research into work and health meant the impact of research was more immediate and easier to demonstrate. Nevertheless, while some respondents optimized the use of new ways of working, others (colleagues and organizations) appeared more reluctant to embrace such non-traditional paradigms of working.

The transition to home working during the paramedic afforded some respondents increased autonomy and greater flexibility in their working lives, freeing up capacity to take on more research work, increased collaboration opportunities and with virtual meetings being more efficient and inclusive. For some, these had positive mental health benefits. However, positive experiences of remote working were not shared by all respondents, with some describing inadequate home-working environments (working in communal areas), feeling isolated from colleagues, lacking ongoing support from team members and reduced opportunities to engage with the wider research community on a regular basis. Others also highlighted difficulties with accessing research resources (e.g. libraries) and fewer professional development opportunities:

Huge disruption to fieldwork, lack of team meetings face to face with many (small and large) aspects or working issues being overlooked or missed because of remote working. Lack of meaningful communication because of remote working/online meetings and a lack of cohesion between the research team.

Furthermore, several respondents expressed the view that remote working resulted in poor team cohesion and made effective communication more challenging among researchers and research groups:

Home working does not suit everyone and that online meetings have limited effect in terms of successfully conducting a study and communicating effectively to the wider team. Clear communication is even more pivotal to the success of team working.

Moreover, several (academic researchers) respondents described the requirement to rapidly transition to remote teaching and learning platforms, and providing emotional support to students whilst also managing personal responsibilities contributed to a significant increase in their workload and time pressure which then created an emotional and physical burden for them to manage. Consequently, these broader pressures had a detrimental impact of their capacity to plan and conduct research.

Others highlighted difficulties with research staff recruitment and retention, the wider disruption experienced in research delivery settings (such as primary care and the wider labour market) along with the increase in workplace anxiety among workers, which all had negative consequences for the delivery and progression of non-Covid-19-related research into work and health. Furthermore, some respondents described physical barriers with accessing and recruiting participants due to restrictions on accessing workplace settings, and the perceived lack of managerial buy-in and support which hampered progression of research into work and health. In other circumstances, workplace intervention delivery and data collection, which took place before and during the onset of the pandemic, has compromised planned pre–post analysis.

Facilitators were also reported. Respondents observed a reduction in the time needed to obtain research governance approvals coupled with increased capacity to set up Covid-19 studies more swiftly than usual contributed to a more streamlined and time-efficient delivery of (Covid-19) research into work and health. However, this was found to contribute to increased time pressure and workload when suspending and subsequently restarting non-Covid-19 studies. For some respondents, this reorientation of work activities, which was intended to release research capacity to support Covid-19 studies, also meant other research work such as analysis and report writing were delayed.

Respondents highlighted a broad range research priorities for work and health which they considered are likely to emerge in response to the Covid-19 pandemic (Table 4).

**Table 4.** Future priorities for research into work and health

• Digital health interventions for managing chronic diseases and embracing more self-management or guided interactive care	• Diversity and equality research
• Effective strategies to deliver more accessible Covid-19 vaccine to key workers	• Employability in disadvantaged groups (patients with disabilities or long-term health conditions)
• Exploring hybrid working and connectivity with remote working	• Health and well-being impacts and impact of staff deployment and management during the crisis
• Health and well-being impacts of dealing with an ongoing crisis in the context of healthcare worker and social care worker vulnerability and resilience	• Impact of Covid-19 on respiratory health, diseases and ill-health and work functioning and participation
• Impact of home/hybrid/lone working on mental health, physical health and disabilities	• Microbial exposures and related health effects
• Pain and work (relationship between employment, pain and musculoskeletal disorders/impact of workplace adjustments for chronic pain)	• Pathogenesis and management of post-Covid-19 syndrome and other long-term conditions and illnesses following Covid-19
• Presenteeism research, its prevention and management	• Mental health and well-being research on research staff
• Self-employment/gig worker access to occupational health	• Real-time evaluation of workplace interventions

In Table 5 respondents proposed several strategies to safeguard the continuation of research into work and health should another unprecedented societal event occur in the future. One respondent also suggested that an overarching research strategic framework which gives due recognition to the importance of research into work and health is needed:

**Table 5.** Strategies to safeguard research into work and health

• Establishing international taskforce groups to oversee the prioritization and coordination of work and health research activities, ensuring representation from interdisciplinary and diverse (including underrepresented) researchers. Establish a strategy for occupational and work and health research.	• Prioritizing and ensuring enablers are in place to support continuation of research activity, including equitable funding for health and work research, improved data availability and establish pathways that allow organizations to support and participate in research.
• Embedding enhanced systems to integrate and utilize research data across healthcare and employment sectors.	• Providing proactive peer mentoring support within the work and health research community, which also includes ensuring a greater level of support is provided to junior researchers whose research portfolios and professional development needs may adversely be impacted by such events.
• Learn from the agility and adaptability of research and research methods used effectively during the pandemic to mitigate risks.

There needs to be a clear occupational health research strategy that doles out equitable research funding alongside those of public and environmental health

## DISCUSSION

Covid-19 has had far-reaching consequences for the development and delivery of research across different research settings and disciplines. However, at the time of the present study there was a paucity of research exploring the impact of the Covid-19 pandemic on the continued development and progression of research into work and health during this period. This study reports on data collected from researchers in this field of research. Most respondents were established academic researchers based in the university setting, with fewer working in healthcare or other sectors. Respondents described a diversity of positive and negative experiences with regard to their capacity to plan and deliver research during the pandemic, highlighted priorities for research into work and health for the future and offered important suggestions for safeguarding work and health research in the future.

A notable strength is that this was the first exploratory study investigating the impact of the Covid pandemic on the development and continuation of research into work and health, and we were able to elucidate some of the practical challenges researchers in the field experienced. The results should be considered however, in light of several limitations. Other comparable studies generated a much higher response rate compared to our study [11]. It is not possible to ascertain the representativeness of the results and there is the potential impact of response bias, particularly since a high proportion of respondents were established researchers and university employed. There could possibly be some disciplines undertaking research in the field of work and health that have not been captured in the study, either due to survey reach or engagement constraints.

Our results were largely consistent with the main findings and observations reported in earlier studies from other fields of research. Specifically, that there were increased opportunities for researchers to expand their traditional areas of research as they explored novel aspects of the Covid-19 virus relating to their field of interest, with this branching out allowing them to engage in new research collaborations with other disciplines [14]. We also found that the adoption of remote working practices by researchers during the pandemic allowed for greater autonomy when undertaking research and seeking better work–life balance, which tended to support more inclusive working arrangements. Additionally, remote (home) working resulted in an increase in productivity and work capacity for some researchers, which is in line with research on the value and importance of flexibility and adaptability at both individual and team level. Specifically, for some, agile ways of working helped to facilitate research delivery and progression, particularly by making it easier for people to participate in research online, and minimized significant disruption caused by the pandemic [4,8,10].

Conversely however, researchers experienced a notable reduction in opportunities to screen and consent participants into studies, coupled with practical difficulties with existing planned research activities. As highlighted in earlier studies, these logistical constraints were often attributed to physical barriers and workplace restrictions designed to mitigate risk (infection prevention) which then restricted access to research settings (including workplaces) and participants [5,8,9]. However, we do acknowledge that earlier studies were specific clinical settings and so may not fully reflect the challenges experienced for work and health researchers.

Despite these challenges and in contrast with other studies, our findings showed that research staff in the field of research into work and health were less adversely impacted with regard to the mandatory requirement to suspend/pause existing non-Covid-19 studies or from the requirement to redeploy research staff to support Covid-19 studies [3,7,10]. We attribute this disparity to the large number of respondents who were employed in a university as opposed to the healthcare sector.

## References

[1] NIHR. DHSC Issues Guidance on the Impact of COVID-19 on Research Funded or Supported by NIHR. UK: National Institute of Health Research, 2020. https://www.nihr.ac.uk/news/dhsc-issues-guidance-on-the-impact-on-covid-19-on-research-funded-or-supported-by-nihr/24469 (15 August 2022, date last accessed).

[2] NIHR. Urgent Public Health COVID-19 Studies 2021. National Institute of Health Research, 2021. https://www.nihr.ac.uk/covid-studies/ (15 August 2022, date last accessed).

[3] Angus DC , Gordon AC, Bauchner H. Emerging lessons from COVID-19 for the US clinical research enterprise. J Am Med Assoc 2021;325:1159–1161. 10.1001/jama.2021.3284 33635309

[4] Fleming TR , Labriola D, Wittes J. Conducting clinical research during the COVID-19 pandemic: protecting scientific integrity. J Am Med Assoc 2020;324:33–34. 10.1001/jama.2020.9286 32463422

[5] Singh JA , Bandewar SV, Bukusi EA. The impact of the COVID-19 pandemic response on other health research. Bull World Health Organ 2020;98:625–631. 33012862 10.2471/BLT.20.257485 PMC7463185

[6] US Food and Drug Administration. FDA Guidance on Conduct of Clinical Trials of Medical Products During COVID-19 Public Health Emergency: Guidance for Industry, Investigators, and Institutional Review Boards. USA: US Dept of Health and Human Services, Food and Drug Administration, Center for Drug Evaluation and Research, Center for Biologics Evaluation and Research, Center for Devices and Radiological Health, Oncology Center of Excellence, Office of Good Clinical Practice, 2020. https://www.fda.gov/media/136238/download (15 August 2022, date last accessed).

[7] Thornton J . Clinical trials suspended in UK to prioritise covid-19 studies and free up staff. Br Med J 2020;368:m1172. 32205354 10.1136/bmj.m1172

[8] Perez T , Perez RL, Roman J. Conducting clinical research in the era of Covid-19. Am J Med Sci 2020;360:213–215. 32690272 10.1016/j.amjms.2020.06.011 PMC7283065

[9] Raghuraman N , Hardy C, Frolova A et al Impact of the COVID-19 pandemic on labor and delivery research operations. Am J Obst Gynecol MFM 2021;3:100443. 34311145 10.1016/j.ajogmf.2021.100443 PMC8302818

[10] Wyatt D , Faulkner-Gurstein R, Cowan H, Wolfe CDA. Impacts of COVID-19 on clinical research in the UK: a multi-method qualitative case study. PLoS One. 2021;16:e0256871. 34464430 10.1371/journal.pone.0256871 PMC8407556

[11] Bratan T , Aichinger H, Brkic N et al Impact of the COVID-19 pandemic on ongoing health research: an ad hoc survey among investigators in Germany. BMJ Open 2021;11:e049086. 10.1136/bmjopen-2021-049086 PMC8649878 34872995

[12] Misra V , Safi F, Brewerton KA et al Gender disparity between authors in leading medical journals during the COVID-19 pandemic: a cross-sectional review. BMJ Open 2021;11:e051224. 10.1136/bmjopen-2021-051224 PMC8282422 34261692

[13] Quak E , Girault G, Thenint MA, Weyts K, Lequesne J, Lasnon C. Author gender inequality in medical imaging journals and the COVID-19 pandemic. Radiology 2021;300:E301–E307. 33724061 10.1148/radiol.2021204417 PMC7983071

[14] Kniffin KM , Narayanan J, Anseel F et al COVID-19 and the workplace: implications, issues, and insights for future research and action. Am Psychol 2021;76:63–77. 32772537 10.1037/amp0000716

[15] O’Connor DB , Aggleton JP, Chakrabarti B et al Research priorities for the COVID-19 pandemic and beyond: a call to action for psychological science. Br J Psychol 2020;111:603–629. 32683689 10.1111/bjop.12468 PMC7404603

[16] Weston D , Parsons V, Ntani G, Rushton L, Madan I. Mixed contact methods to improve response to a postal questionnaire. Occup Med (Lond) 2017;67:305–307. 28371932 10.1093/occmed/kqx032

[17] The British Psychological Society. Ethics Guidelines for Internet-Mediated Research 2021. The British Psychological Society, 2021. https://www.bps.org.uk/guideline/ethics-guidelines-internet-mediated-research (15 August 2022, date last accessed).

[18] Kaplowitz MD , Hadlock TD, Levine R. A comparison of web and mail survey response rates. Public Opin Q 2004;68:94–101.

